# Geriatric Trauma – A Rising Tide. Assessing Patient Safety Challenges in a Vulnerable Population Using Norwegian Trauma Registry Data and Focus Group Interviews: Protocol for a Mixed Methods Study

**DOI:** 10.2196/15722

**Published:** 2020-04-30

**Authors:** Mathias Cuevas-Østrem, Olav Røise, Torben Wisborg, Elisabeth Jeppesen

**Affiliations:** 1 Department of Research Norwegian Air Ambulance Foundation Oslo Norway; 2 Norwegian Trauma Registry Division of Orthopaedic Surgery Oslo University Hospital Oslo Norway; 3 Faculty of Health Sciences University of Stavanger Stavanger Norway; 4 Institute of Clinical Medicine Faculty of Medicine University of Oslo Oslo Norway; 5 Norwegian National Advisory Unit on Trauma Division of Emergencies and Critical Care Oslo University Hospital Oslo Norway; 6 Anaesthesia and Critical Care Research Group Faculty of Health Sciences University of Tromsø - The Arctic University of Norway Tromsø Norway

**Keywords:** major trauma, multiple trauma, aging, older adults, elderly, brain injuries, traumatic, geriatric, epidemiology, trauma registries, quality of health care, injury severity score

## Abstract

**Background:**

Elderly trauma patients constitute a vulnerable group, with a substantial risk of morbidity and mortality even after low-energy falls. As the world’s elderly population continues to increase, the number of elderly trauma patients is expected to increase. Limited data are available about the possible patient safety challenges that elderly trauma patients face. The outcomes and characteristics of the Norwegian geriatric trauma population are not described on a national level.

**Objective:**

The aim of this project is to investigate whether patient safety challenges exist for geriatric trauma patients in Norway. An important objective of the study is to identify risk areas that will facilitate further work to safeguard and promote quality and safety in the Norwegian trauma system.

**Methods:**

This is a population-based mixed methods project divided into 4 parts: 3 quantitative retrospective cohort studies and 1 qualitative interview study. The quantitative studies will compare adult (aged 16-64 years) and elderly (aged ≥65 years) trauma patients captured in the Norwegian Trauma Registry (NTR) with a date of injury from January 1, 2015, to December 31, 2018. Descriptive statistics and relevant statistical methods to compare groups will be applied. The qualitative study will comprise focus group interviews with doctors responsible for trauma care, and data will be analyzed using a thematic analysis to identify important themes.

**Results:**

The project received funding in January 2019 and was approved by the Oslo University Hospital data protection officer (No. 19/16593). Registry data have been extracted for 33,344 patients, and the analysis of these data has begun. Focus group interviews will be conducted from spring 2020. Results from this project are expected to be ready for publication from fall 2020.

**Conclusions:**

By combining data from the NTR with interviews with doctors responsible for treatment and transfer of elderly trauma patients, we will provide increased knowledge about trauma in Norwegian geriatric patients on a national level that will form the basis for further research aiming at developing interventions that hopefully will make the trauma system better equipped to manage the rising tide of geriatric trauma.

**International Registered Report Identifier (IRRID):**

PRR1-10.2196/15722

## Introduction

### Background

Many high- and middle-income countries around the world face the same demographic changes: people are living longer, birth rates are decreasing, and, consequently, elderly people constitute a rapidly growing proportion of the population [1,2]. The elderly often live independent and active lives despite chronic diseases and frailty and can sustain severe injury from even minor trauma [3-5]. Statistics Norway estimates that within 15 years, more people living in Norway will be aged above 65 years than below 20 years, for the first time [6]. The same report projects that by 2060, the number of Norwegians aged above 70 years will be more than double compared with the number in 2018 [6]. Consequently, there is an increase in the number of geriatric trauma patients, and the geriatric trauma population has been described as a rising tide [7].

Trauma is one of the leading causes of mortality and morbidity worldwide and in all age groups [8,9]. In Norway in 2016, the most common injuries across all ages occurred in the extremities (38.3%), head (35.4%), chest (29.5%), and spine (24.3%) [10]. Geriatric trauma patients have higher mortality rates than younger patients, adjusted for the same severity of trauma, and head injury is the leading cause of death [11-13]. Risk factors associated with a poor outcome for this group include age, pre-existing medical conditions, anticoagulant use, frailty, and altered physiological response to trauma [14-20]. Hence, geriatric trauma patients are a vulnerable group.

There is an evident shift in the epidemiology of major trauma: what used to be the disease of young men in high-energy accidents is now becoming the disease of elderly patients, where the primary mechanism of injury (MOI) is falling from less than 2 meters [21,22]. Major trauma is usually defined using the Injury Severity Score (ISS) or New Injury Severity Score (NISS), and the most common threshold is ISS >15 [23]. It has been questioned if this is too high for geriatric trauma patients, as the frail elderly might have significant morbidity and mortality even at low thresholds [24]. The age of 65 years is widely used as a cutoff for defining geriatric trauma [16,22,25-27].

### Characteristics of the Geriatric Trauma Population

A 2017 report from the UK Trauma Audit and Research Network gives new and thorough insight into the characteristics of geriatric major trauma patients [22]. Some of the central findings were that over 60% of trauma patients aged 70 years and above are injured indoors, the head was the most commonly injured body region, older people admitted to hospitals had a low trauma team activation rate, and the grade of the most senior clinician treating the patients on arrival decreased with increasing patient age [22]. Low-energy trauma attracts little attention.

A geriatric trauma patient is not simply an injured old adult. Pharmacological and age-related physiological alterations in different organ systems affect the way the geriatric patient responds to both disease and injury [28]. Among the changes relevant for trauma care is that geriatric patients are often frail, meaning they have low physiological reserves [14]; they present with a higher Glasgow Coma Scale (GCS) score compared with younger patients with the same injury severity [29]; the threshold for hypotension is suggested to be 110 mm Hg, not 90 mm Hg [30,31]; and with increased age, the use of physiology-altering medications such as beta antagonists or anticoagulants increases. This might mask the severity of injury as the vital signs resemble what is considered to be within normal range values for adults. As a consequence, an injured elderly patient might seem less injured when standard triage tools are used, and this is reflected in the high rate of undertriage for geriatric major trauma patients [3,32,33]. Undertriage increases the risk of not being treated at the right level of care at the right time and can, subsequently, increase the risk of mortality [32].

Major trauma is a time-critical event; hence, disposing the right resources at the right time without unjustifiable delay is crucial. Paradoxically, it is the elderly patients—the ones with the least physiological reserves—who get delayed treatment [22,34,35]. Both Advanced Trauma Life Support (ATLS) and the Eastern Association for the Surgery of Trauma geriatric trauma guidelines advocate for an aggressive treatment approach until otherwise decided [27,28]. Early and aggressive treatment is shown to increase survival rates in older trauma patients [36].

### Traumatic Brain Injury

Traumatic brain injury (TBI) is one of the leading causes of trauma-related deaths [37]. Antiplatelet and anticoagulant drugs are frequently used in the geriatric trauma population, a risk factor for acute intracranial bleeding following head injury. A computed tomography (CT) head scan is needed to detect bleedings, and this can be done in all acute care trauma hospitals in Norway. In cases of moderate-to-severe TBI, the acute care trauma hospital can contact the neurosurgical department in the regional trauma center for clinical guidance and assessment of patient transfer. Experience from clinical practice nationally and internationally shows that the transfer of elderly trauma patients with head injury to a neurosurgical facility from an acute care trauma hospital is a challenge 35. We believe that there are more factors than just injury, severity, and national transfer criteria that determine whether patients are transferred from an acute care trauma hospital to a trauma center with neurosurgical facilities. We believe that possible factors are age, comorbidities, activities of daily life functions, prognosis, limitations in ward capacity, limitations in what the neurosurgical intervention can offer to improve prognosis, and limited time before it is too late to intervene, along with culture and an expectation of a negative outcome. This will be explored in this project.

### Norwegian Trauma System

The 2016 National Trauma Plan for Norway provides requirements for all services in the national trauma system—from prehospital care to rehabilitation [38]. Norway has 2 hospital levels treating trauma patients; 34 acute care trauma hospitals and 4 trauma centers. Acute care trauma hospitals are spread out around the country, and trauma centers are regional university hospitals. All acute care trauma hospitals offer general surgical and orthopedic services and are capable of stabilizing severely injured patients before transferring them to trauma centers, if necessary, but do not offer neurosurgery, intervention radiology (except for a few), and other specialized services. The trauma centers offer all medical specialties, including neurosurgery, and are capable of managing all types of injuries [39]. The annual number of patients meeting the inclusion criteria of the Norwegian Trauma Registry (NTR; see Methods) is approximately 8000 [40].

NTR is a national medical quality registry that has been operational from January 2015. The objective of the registry is to monitor trauma treatment in Norway and to contribute to increased treatment quality. All acute care trauma hospitals and trauma centers in Norway report to the registry. These hospitals have certified registrars who register data from injury to rehabilitation after the Utstein template and classify all injuries according to the Abbreviated Injury Scale (AIS) and calculate ISS and NISS [41,42]. All patients receive written information about the registry, including the opportunity to access the data recorded and to deny registration.

### Aims and Objectives

The aims of this project are to investigate whether patient safety challenges exist for elderly trauma patients in Norway and to identify risk areas that will facilitate further work to safeguard and promote quality and safety in the Norwegian trauma system. A total of 3 retrospective cohort studies and 1 qualitative interview study will be conducted. The results of each study will be published in peer-reviewed medical journals.

The specific objectives of the project are as follows:

1. To assess whether injured elderly Norwegian patients (65 years) are given different emergency trauma care compared with younger patients.
2. To explore explanations for potential differences in the quality of trauma care between age groups in the emergency part of the trauma chain.

The quantitative studies aim to achieve the following:

- Determine the characteristics of geriatric trauma patients in Norway and compare this group with the Norwegian adult population and results from comparable international publications.
- Describe differences between the adult and elderly general and TBI populations in Norway regarding injury severity, MOI, 30-day mortality, hospital level of care, transport methods, emergency interventions, radiological examinations, and physiological variables.

The qualitative interview study aims to achieve the following:

- Explore factors that may affect transfer decisions for geriatric patients with TBI.

## Methods

### Study Design

**Figure 1:**
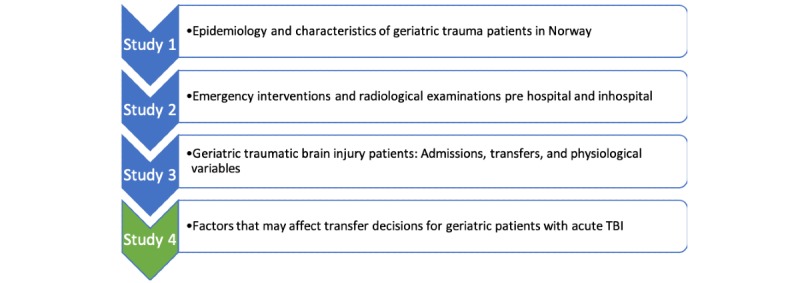
An overview of the four studies included in the project. TBI: traumatic brain injury.

The *Geriatric Trauma—Assessing Patient Safety* project applies a mixed methods design, and it consists of 3 quantitative retrospective cohort studies using data from the NTR and 1 qualitative interview study focusing on the management of patients with acute TBI. The 4 studies included in the project are presented in Figure 1. The use of both qualitative and quantitative methods provides a deeper understanding of the processes involved in the care of elderly TBI patients and can increase the understanding of causative factors regarding the management of this group. The qualitative study provides an extra layer of information that will help interpret the quantitative data on TBI so that it can be better used in improving the trauma system.

### Study Setting

The main study setting is the prehospital and emergency department part of the Norwegian trauma system. Data from the NTR are collected from all acute care trauma hospitals and trauma centers in Norway, which has a population of about 5.3 million inhabitants spread out over vast distances with a mix of urban and rural areas. For the qualitative study, we seek to include a sample with representatives from at least two Norwegian health regions.

### Study Participants: Data From the Norwegian Trauma Registry

The NTR was searched to identify all trauma patients included in the registry from January 1, 2015, to December 31, 2018. A total of 33, 344 patients were included in the registry, of which 22,603 were aged between 16 and 64 years and 6334 were 65 years or older. A total of 3735 elderly patients had NISS above or equal to 9. The eligibility criteria for the registry are presented in Textboxes 1 and 2.

**Inclusion criteria for the Norwegian Trauma Registry.**

- All patients admitted with trauma team activation (TTA) on arrival to the emergency department in all acute care trauma hospitals and trauma centers in Norway, irrespective of Injury Severity Score and New Injury Severity Score (NISS)
- All patients treated at an acute care trauma hospital or trauma center in Norway, without TTA, with one or more of the following injuries:
  Penetrating injury to the head, neck, torso, or extremities proximal to the elbow or knee
  Head injury with abbreviated injury score (AIS) ≥3
  NISS >12
- All patients with trauma-related deaths at the site of trauma or during transportation to the hospital, who are not referred to the hospital, but where prehospital management or treatment is initiated

**Exclusion criteria for the Norwegian Trauma Registry.**

- Patients with chronic subdural hematoma, without other trauma-related injuries
- Patients with injuries from drowning, inhalation, hypothermia, and asphyxia without concomitant trauma
- Patients who die on scene without the activation of prehospital resources

### Quantitative Registry-Based Retrospective Cohort Studies

The 3 quantitative studies are all registry-based retrospective cohort studies focusing on (1) epidemiology and characteristics, (2) emergency interventions and radiological examinations, and (3) TBI in the Norwegian geriatric trauma cohort. The specific outcome measures for each study are presented in Table 1, and a full overview of the variables extracted from the registry is presented in Multimedia Appendix 1.

Study number three focuses on elderly TBI patients, a particularly vulnerable patient group with high morbidity and mortality [37]. The severity of TBI can be defined using different measures. AIS is an international classification system defining all injury types according to severity where 1 is minor and 6 is maximal and currently untreatable [42]. AIS ≥ 3 is recognized as moderate-to-severe head injury.. GCS at presentation is one of the major factors directing neurosurgical decision making, traditionally classifying TBI into mild (GCS 13-15), moderate (GCS 9-12), and severe (GCS <8). Recent evidence suggests that GCS is not as sensitive for detecting TBI in the elderly, so we will do analyses for both parameters [29]. In addition, GCS is the only measure of the two with prehospital value. Patients admitted with a low GCS not caused by head trauma will be excluded from these analyses.

**Table 1:** Overview of the quantitative studies.

Characteristics	Study 1	Study 2	Study 3
Aims	Describe the Norwegian geriatric trauma population and assess differences in demographic and epidemiological characteristics between age groups Assess 30-day mortality Identify injury mechanism differences between age groups Assess differences in the prehospital and inhospital levels of care between age groups	Assess differences in the proportion of emergency interventions (prehospital and inhospital airway management and pneumothorax decompression) and radiological examinations (inhospital) performed on elderly and younger patients Assess differences in emergency interventions and radiological examinations performed on elderly and younger patients on the basis of clinical findings	Assess differences in admission rates and transfer rates to trauma centers with neurosurgical services for patients in different age groups with moderate-to-severe traumatic brain injury Assess differences between age groups in transport method (car or air ambulance) for patients with same degree of injury severity Assess differences in physiological variables between age groups, both prehospital and at admission (systolic blood pressure, respiratory rate, Glasgow Coma Scale score, and body temperature) for patients with the same degree of injury severity
Hypothesis	Younger patients suffer primarily from injury due to high-energy trauma, and elderly patients suffer primarily from injury due to low-energy trauma Younger patients have higher admission rates to trauma centers than the elderly for similar injury severity Younger patients have higher transfer rates to trauma centers than the elderly for similar injury severity	Prehospital personnel use the same algorithm in decision making in both elderly and younger patients, that is, there is no discrimination in how elderly and younger patients with the same vital signs are treated The elderly population is expected to have same frequencies of examinations and interventions as the younger patients for the same severity of injuries, both prehospital and in the emergency room	Younger patients have higher admission rates to trauma centers than the elderly Younger patients have higher transfer rates to trauma centers than the elderly Younger patients are more often transported by air ambulance than the elderly
Outcome measures	Primary: 30-day mortality Secondary: Age, gender, mechanism of injury, blunt or penetrating trauma, Abbreviated Injury Scale, Injury Severity Scale, New Injury Severity Scale Location of injury Time from injury to admission Transport method Level of prehospital and inhospital care Interventions given prehospitally and in the emergency department Trauma team activation Level of care at admission and discharge Length of stay	Primary: Number and type of radiological examinations and emergency interventions (frequencies) Secondary: Time to examination (x-ray; thorax, pelvis, and computed tomography) Physiological variables	Primary: 30-day mortality Secondary: Admissions to acute care trauma hospitals and trauma centers Transfers to higher level of care Transport methods Physiological variables Interventions given prehospitally and in the emergency department

### Data Analysis

All injured adult patients admitted to a Norwegian hospital and registered in the NTR in the period January 1, 2015, to December 31, 2018, will be included in the analysis. Trauma registry data will be analyzed using descriptive statistical methods and other relevant statistical methods to compare adult (aged 16-64 years) with elderly (aged 65 years) trauma patients, as described below. Data might also be analyzed to compare subgroups, for example, 10-year age intervals, if the data allow it. Data will be reported following the Strengthening the Reporting of Observational Studies in Epidemiology statement checklist. Categorical variables will be analyzed using a Pearson chi-square test, continuous variables will be analyzed with normal score distribution using *t* tests, and skewed distributions will be analyzed using the Mann-Whitney *U* test. We will consider using the Fisher exact test for smaller subgroups. We will also consider doing a logistic regression analysis. The strength of association will be reported as an odds ratio with 95% CI. Low statistical power because of small groups and few events could result in some significant differences with broad 95% CIs. The correlation between the age groups is planned to be tested with a Spearman rank correlation test. We consider our study as explorative, and the significance level will therefore be kept at *P*<.05. The analyses would be performed by using SPSS version 25 or higher (IBM SPSS Statistics for Mac, IBM Corporation).

All data will be handled and saved in a secured data server administered by the Norwegian Air Ambulance Foundation. All data will be unidentifiable when sharing between the authors and in the analysis and presentations. Data will be stored for 5 years after the project is finished.

### Study 4: Qualitative Interview Study Addressing Factors That May Affect and Explain Transfer Decisions for Geriatric Patients With Acute Traumatic Brain Injury

#### Participants

A sample of participants for the focus group interviews will be recruited from doctors responsible for the treatment and transfer decisions for head trauma patients. We seek to include participants with the following characteristics:

- Acute care trauma hospital team leaders: Responsible for initial evaluation, transfer evaluation, and continued care in case the patient is not transferred. We seek registrars or consultants with more than 1 year of experience as a trauma team leader and trained in the ATLS principles according to the requirements in the national trauma plan. The subjects should preferably have experienced at least one case of a geriatric trauma patient where head trauma was the main reason for discussing transfer.
- Neurosurgeons in trauma centers: Taking part in decision making on accepting the patient for transfer or not, being responsible for all neurosurgical interventions, monitoring, and care in a neurosurgical ward. We seek registrars or consultants with more than 1 year of experience in on-call decision making, assessing patients for transfer to their respective hospitals.

A priori, it is estimated that 4 focus group interviews will be sufficient, but data acquisition will continue until saturation is reached. The interviews will be conducted separately (mono-professional) to reveal possible professional differences. The groups will be recruited using a combination of the snowball sampling method to reach out to a wide network, purposive sampling to include doctors with first-hand experience with geriatric head trauma and working locations in different health regions, and convenience sampling to conduct interviews in regional or national forums. All participants will receive written and oral information about the purpose of the study. We will also obtain informed consent. Before starting the interview, they will be informed that they are discussing factors affecting management and transfer decisions in patients with TBI. The interviewer will use an interview guide with open-ended questions to ensure that the relevant subjects are covered. This will cover themes such as priorities and ethical considerations, patient-related factors emphasized in the decision-making process, guidelines, attitudes, culture, and interventions.

#### Analysis

The interviews will be audio-recorded and transcribed verbatim. The data found in the interviews will be categorized and analyzed using thematic analysis as described by Braun and Clark [43]. Each interview will be coded by at least two analysts who will read the transcripts and, if appropriate, listen to the audio recordings to ensure the proper meaning is captured. The analysts will generate codes and sort these into themes. Coding disparities or uncertainties will be discussed with additional researchers in the group.

### Ethical Considerations

Research will be conducted according to the ethical guidelines of the Helsinki declaration. The study protocol is approved by the Oslo University Hospital data protection officer, which is responsible for the Norwegian Trauma Registry (No. 19/16593).

## Results

Registry data have been extracted on 33,344 patients, and the analysis of data for study number one is ready to be performed. Anticipated findings are that the Norwegian geriatric trauma population shows a number of similar characteristics as described in papers from comparable Western populations (e.g. the Netherlands, United Kingdom, and Australia) but that the proportion of geriatric trauma patients is smaller than that in countries with a larger elderly population, for example, Japan [22,44-46]. The next steps will be to work parallelly on the manuscript on study 1 and on conducting interviews for study 4. Studies 2 and 3 will be conducted subsequently. The project plan has been presented in relevant forums in Norway and Europe. Results from each study will be published in peer-reviewed medical journals from 2020.

## Discussion

### Principal Considerations

The vulnerable population of geriatric trauma patients is increasing in number. It is a group with clinically challenging characteristics, such as comorbidity, polypharmacy and frailty, and a high risk of undertriage. As major trauma shifts from being a disease of the young to a disease of the elderly injured in low-energy accidents, substantial patient safety risks may exist, for example, differences in the level of care between adults and elderly patients. To our knowledge, no study has been conducted in Norway by using national data assessing such differences in trauma care.

Kirkman et al [35] published a paper that raises the following central question: “Do elderly head injuries do worse because of a self-fulfilling prophecy of poorer management?” They found that the time from admission to CT head imaging and the likelihood of not being transferred to a center with acute neurosurgical care facilities increased with age. Another study from Utter et al [34] found that geriatric trauma patients have delayed transfer to a neurocenter in a level I trauma center [34]. Little is known about which factors affect the decisions that lead to this. Negative attitudes toward elderly patients and an expectation of a poor outcome might lead to a passive, observing role and low treatment ambitions, and this will be addressed in this project.

In 2019, a paper about geriatric trauma patients from the largest trauma center in Norway was published, showing that mortality increased with age and was inversely related to the probability of trauma team activation on arrival [13]. Moreover, Australian, Dutch, British, and Japanese papers published in the recent years give a thorough overview of the characteristics of geriatric trauma populations in comparable countries [22,44-46]. Whether the total Norwegian trauma population shares some of these characteristics is not known.

### Strengths and Limitations

The project employs a mixed methods design, where possible patient safety challenges of the geriatric trauma population will be assessed through 4 studies. The mixed methods design is one of the project’s strengths, as the qualitative methodology brings forward information that the registry data cannot provide and makes the interpretation of the retrospective data more reliable when it should be translated into clinical practice. Another strength with this project is how it focuses particularly on the potential patient safety challenges of elderly trauma patients. As far as we know, it is the first project on geriatric trauma patients where patient safety is the overarching theme. A high generalizability to other trauma systems is expected, given the similarities between demographical changes and trauma systems in many high-income countries. Limitations are inherent to the retrospective design of the quantitative studies, with risk of bias and the fact that causal factors cannot be explored.

### Conclusions

With the rising tide of geriatric trauma as a background, this research will have a societal impact. If there are differences between adult and elderly trauma patients, it is important to know to make sound decisions in the future. For example, if geriatric trauma patients are found to be systematically treated at a lower level of trauma care, it will be important to document this, and the next step will be to examine why these differences exist. Findings regarding characteristics and physiological responses will possibly support international studies, and ours will be the first study to assess this in the Norwegian population. With the rising tide of geriatric trauma fast approaching, we want to investigate differences in trauma care between age groups in the Norwegian population and evaluate if patient safety risks exist for geriatric trauma patients.

## Appendix

Multimedia Appendix 1 List of extracted variables from the Norwegian Trauma Registry.

## Abbreviations

AIS: Abbreviated Injury Scale
ATLS: advanced trauma life support
CT: computed tomography
GCS: Glasgow Coma Scale
ISS: Injury Severity Score
MOI: mechanism of injury
NISS: New Injury Severity Score
NTR: Norwegian Trauma Registry
TBI: traumatic brain injury
