# Supplementary material for: Geriatric Trauma – A Rising Tide. Assessing Patient Safety Challenges in a Vulnerable Population Using Norwegian Trauma Registry Data and Focus Group Interviews: Protocol for a Mixed Methods Study
Source: JMIR Res Protoc. 2020 Apr 30;9(4):e15722. doi: 10.2196/15722 (PMC7226039; doi:10.2196/15722)
Supplement: Multimedia Appendix 1 [file resprot_v9i4e15722_app1.docx]

# Appendix 1

## List of variables extracted from the Norwegian Trauma Registry

All variables are available at <http://www.ntr-definisjonskatalog.no>

| **Description of data variable** |  | **Variable name** |
| --- | --- | --- |
| Unique number for each registered trauma event. If a patient has more than one registered event in the database a unique Trauma-ID number will be given for each event. | 6.1.4 | pt_id_ntr |
| Gender |  | gender |
| Number of the municipality where the injury occurred (Based on Statistics Norway’s list) | 7.1.3 | acc_municip_code |
| Patient pre-injury comorbidity as defined by ASA score | 7.1.5 | pt_asa_preinjury |
| **Chapter 7.2: Was the injury a transport accident?** | 7.2.1 | acc_transport |
| **Chapter 7.3: Other types of injuries:**   - Fall-related injury? - Violence-related injury? - Self-inflicted injury? - Work-related injury? - Recreational injury? - Fire or inhalation injury? - Other? | 7.3.1  7.3.2  7.3.3  7.3.4  7.3.5  7.3.6  7.3.7 | acc_fall  acc_violence  acc_self_inflict  acc_work  acc_sprt_recreat  acc_fire_inhal  acc_other |
| **Chapter 7.4: Injury type and mechanism:**   - Blunt injury? - Penetrating injury? - What was the dominating injury? - What was the injury mechanism? - What was the intention? | 7.4.1  7.4.2  7.4.3  7.4.4  7.4.5 | inj_blunt  inj_penetrating  inj_dominant  inj_mechanism  inj_intention |
| **Chapter 8.1: Prehospital, Emergency Medical Communication Centre (EMCC)**   - Transport type? - Time from alarming EMCC to resources on scene - Time from resource arrival on scene to start of patient transport: On-scene time | 8.1.4  8.1.8  8.1.9 | pre_transport  dt_alarm_scene  dt_ scene_depart |
| **Chapter 8.2: Prehospital physiological status**   - Prehospital systolic blood pressure? - Prehospital respiratory rate? - Prehospital GCS sum? | 8.2.1  8.2.3  8.2.8 | pre_sbp_valu  pre_rr_value  pre_gcs_sum |
| **Chapter 8.3: On-scene management**   - Were airways managed on-scene? - Type of prehospital airway management? - Was prehospital pneumothorax relief given? - Type of prehospital ptx relief? - Highest level of prehospital provider - Was the patient directly transported to dedicated trauma hospital? | 8.3.1  8.3.2  8.3.3  8.3.4  8.3.6  8.3.7 | pre_intubated  pre_intub_type  pre_ptx_relief  pre_ptx_relief_type  pre_provider  pre_transport_traumacentre_direct |
| **Chapter 9.1: Arrival Emergency Dept. (ED)**   - Time from alarming EMCC to arrival in ED - Time from departure scene of injury to arrival in ED - Was trauma team activated on arrival? | 9.1.2  9.1.3  9.1.4 | dt_alarm_hosp  dt_scene_ed  ed_tta |
| **Chapter 9.2: ED physiological status**   - ED systolic blood pressure? - ED respiratory rate? - ED GCS sum? | 9.2.1  9.2.3  9.2.8 | ed_sbp_value  ed_rr_value  ed_gcs_sum |
| **Chapter 9.4:** **Radiology**   - Was chest x-ray (CXR) taken in ED? - Date and time for first CXR? - Was pelvic x-ray taken in ED? - Date and time for first pelvic x-ray? - Was CT scan performed? - Time from arrival to first CT scan? | 9.4.1  9.4.2  9.4.3  9.4.4  9.4.5  9.4.6 | xray_chst  xray_chst_dtg  xray_pelv  xray_pelv_dtg  ed_ct  ed_first_ct_time_exact |
| **Chapter 9.5: Interventions in the ED**   - Was the patient intubated? - Was a chest drain inserted? | 9.5.1  9.5.2 | ed_intubated  ed_chest_drain |
| **Chapter 10: Results**   - To where was the patient discharged? - Hospital length of stay? - Did the patient die within 30 days? - Time interval from injury to death occurred | 10.3.2  10.3.5  10.4.1  10.4.4 | hosp_dischg_dest  hosp_los_days  res_survival  dt_inj_death |
| **Chapter 11: Injury registration**   - Abbreviated Injury Scale (AIS) codes - Automatically calculated ISS - Automatically calculated NISS | 11.1.2  11.2.1  11.2.2 | inj_ais  inj_iss  inj_niss |
